# Genetic Evolutionary Analysis and Characterization of Bovine Viral Diarrhea Virus in Gansu Province, China

**DOI:** 10.3390/v18060598

**Published:** 2026-05-25

**Authors:** Cong Li, Shandian Gao, Yongli Mo, Zhijie Liu, Guangqing Zhou, Xiaoan Cao, Jijun He, Ligang Yuan, Youjun Shang

**Affiliations:** 1 College of Veterinary Medicine, Gansu Agricultural University, Lanzhou 730070, China; licong05092026@163.com (C.L.); 15593887725@163.com (Z.L.); 2 State Key Laboratory for Animal Disease Control and Prevention, Lanzhou Veterinary Research Institute, Chinese Academy of Agricultural Sciences, Lanzhou 730046, China; gaoshandian@caas.cn (S.G.); moyongli26@163.com (Y.M.); zhouguangqing@caas.cn (G.Z.); caoxiaoan@caas.cn (X.C.); hejijun@caas.cn (J.H.)

**Keywords:** bovine viral diarrhea virus (BVDV), RT-PCR, phylogenetic tree, virus isolation, virus identification

## Abstract

This study aimed to investigate the current epidemiology and genetic evolution of bovine viral diarrhea virus (BVDV) on cattle farms in Gansu Province, China, between 2021 and 2025. A total of 749 samples from 62 farms across 14 cities and prefectures in Gansu were tested. The overall BVDV positivity rate was 19.89%, determined by amplification of the 5′-UTR and N^pro^ regions. Seven subtypes were identified: BVDV-1a, -1b, -1d, -1m, -1v, -1u, and -2a. BVDV-1u was the predominant subtype (59.06%), followed by BVDV-1v (13.42%). The greatest subtype diversity was observed in Jinchang City and Gannan Tibetan Autonomous Prefecture. A non-cytopathic BVDV-1v strain, designated YC-2025-Gansu2 (GenBank accession no. PV945812.1), was isolated. This study expands the BVDV subtype database for Gansu and supports the development of subtype-specific prevention strategies in the region.

## 1. Introduction

Bovine viral diarrhea (BVD) is a major contagious disease in cattle, characterized by diarrhea, fever, and coughing. In pregnant cows, it can also cause abortion or fetal malformations. The causative agent, bovine viral diarrhea virus (BVDV), belongs to the family *Flaviviridae*, genus *Pestivirus*, and has been reported in 88 countries worldwide [1,2]. Based on genetic differences, BVDV is classified into three main species: BVDV-1, BVDV-2, and BVDV-3 [3]. To date, 23 subtypes from 1a to 1w have been identified for BVDV-1 [4,5,6,7], five subtypes from 2a to 2e have been described for BVDV-2 [8,9], and three subtypes for BVDV-3 [8,10,11]. BVDV-1 is the most prevalent type worldwide, with particularly high circulation in Asia [12,13,14,15,16]. In China, reported subtypes include 1a, 1b, 1c, 1d, 1m, 1o, 1p, 1q, 1u, 1v, 2a, and 2b [7]. BVDV isolates are also divided into two biotypes: cytopathic (CP) and non-cytopathic (NCP) [17]. In China, most clinical isolates are NCP, which is mainly associated with persistent infection and immunosuppression, while the CP biotype is more commonly linked to mucosal disease [12].

The 5′UTR of most BVDV genomes is relatively conserved and is commonly used for genotyping [18,19,20]. Other genomic regions, including the N^pro^ [21] and E2 [5] coding sequences, are also used for typing. Several studies have shown that combining multiple regions, such as NS4B with NS5B, or 5′UTR with E2, improves subtype classification [4,5,6,7].

To investigate the epidemiology and molecular evolution of BVDV in Gansu Province, 749 samples were collected from 14 cities between 2021 and 2025. BVDV was detected by RT-PCR, and viral genotypes were determined by phylogenetic analysis. Virus isolation and identification were performed on positive samples. This study provides a scientific basis for BVDV prevention, control, and eradication on cattle farms in Gansu Province.

## 2. Materials and Methods

### 2.1. Sample Collection

A total of 749 samples were collected from 62 cattle farms across 14 cities and prefectures in Gansu Province between 2021 and 2025. The sample types included 713 bovine serum samples, 18 fecal samples, 2 spleen tissue specimens, 2 intestinal tissue specimens, and 14 nasal swab samples. Each sample was obtained from an individual animal on each farm.

### 2.2. Detection and Analysis of BVDV

A total of 749 samples were screened for BVDV by conventional RT-PCR targeting the 5′UTR and N^pro^ regions using two primer sets (Table 1). All primers were synthesized by Sangon Biotech (Shanghai, China).

Total RNA was extracted using the QIAamp RNeasy Mini Kit (Qiagen, Hilden, Germany). For conventional RT-PCR, a 50 µL reaction mixture contained 25 µL of 2× One Step Reaction Solution, 2 µL of One Step Enzyme Mix, 2 µL each of forward and reverse primers, 3 µL of RNA template, and RNase-free water to a final volume of 50 µL. Thermal cycling conditions were: reverse transcription at 50 °C for 30 min; initial denaturation at 95 °C for 3 min; 35 cycles of 95 °C for 30 s, 55 °C for 30 s, 72 °C for 50 s; and a final extension at 72 °C for 10 min. PCR products were separated on a 1.0% agarose gel, and target bands were purified and sequenced by Sangon Biotech.

### 2.3. Antigen Testing

Serum samples positive by nucleic acid detection were further tested for BVDV antigen using the IDvet BVDV P80 protein ELISA antigen detection kit (IDvet, Grabels, France) following the manufacturer’s instructions. Absorbance was measured at 450 nm using a microplate reader (Thermo Fisher Scientific, Waltham, MA, USA). The assay was considered valid only when the mean optical density of the positive control exceeded 0.500 and the positive/negative control OD ratio was greater than 3. Sample results were calculated as S/P = (OD_sample_ − OD_NC_)/(OD_PC_ − OD_NC_) × 100%. Samples with S/P ≥ 40% were classified as positive, while those with S/P < 40% were considered negative.

### 2.4. Phylogenetic Analysis

The obtained 5′UTR and N^pro^ sequences were aligned with reference strains from GenBank. Phylogenetic analysis was performed using MEGA 11.0 (Mega Limited, Auckland, New Zealand) with the neighbor-joining method and the Kimura 2-parameter model. Branch support was assessed with 1000 bootstrap replicates.

### 2.5. Isolation and Identification of BVDV

#### 2.5.1. Isolation of BVDV

Each of the five BVDV antigen-positive serum samples was individually processed for virus isolation. Each sample was filtered through a 0.22 μm membrane to remove bacteria. Then, 300 μL of the filtrate from each sample was inoculated into confluent MDBK cell culture flasks. After incubation at 37 °C with 5% CO_2_ for 2 h, the inoculum was replaced with DMEM maintenance medium containing 2% fetal bovine serum. Cultures were observed daily for cytopathic effects. After 5 days, cells were harvested and subjected to three freeze–thaw cycles at −80 °C, followed by five consecutive blind passages. Harvested material from each passage was stored at −80 °C for subsequent use.

#### 2.5.2. Characterization of Isolated Virus

RNA was extracted from virus cultures at passages three to five and tested by RT-PCR. Virus from the fifth passage was inoculated onto MDBK cells on confocal dishes. After 12 h, cells were fixed with 4% paraformaldehyde, stained with FITC-conjugated BVDV-specific antibodies, counterstained with DAPI, and observed under a fluorescence microscope. Separately, supernatant from the fifth-passage culture was concentrated by centrifugation at 32,000 rpm for 2.5 h at 4 °C, negatively stained with 2% phosphotungstic acid, and examined under a transmission electron microscope.

### 2.6. Whole-Genome Sequencing

RNA was extracted from the fifth-passage viral culture and sent to Shanghai Tanpu Biotechnology Co., Ltd. (Shanghai, China) for next-generation sequencing to obtain the complete viral genome.

### 2.7. Sequence and Structure Analysis

#### 2.7.1. Recombination Analysis of BVDV Complete Genome

Potential recombination events were analyzed using RDP4 (Recombination Detection Program, version 4.101; http://web.cbio.uct.ac.za/~darren/rdp.html, accessed on 23 April 2026) [22]. The complete genome nucleotide sequences of all BVDV strains were aligned using ClustalW prior to analysis. Seven methods implemented in RDP4 were used for recombination detection: RDP, GENECONV, BootScan, MaxChi, Chimaera, SiScan, and 3Seq [22,23,24,25,26,27,28]. Following the server’s default parameters, the window size was set to 200 bp, the step size to 20 bp, the number of bootstrap replicates for BootScan to 1000, and the P-value threshold to 0.05. Only recombination events detected by at least three of the seven methods were considered statistically reliable.

#### 2.7.2. Sequence-Structure Analysis of the E2 Protein

Multiple E2 protein sequences were aligned using ClustalW. The alignment was visualized using ESPript 3.2 (available at: https://espript.ibcp.fr, accessed on 31 March 2026) [29]. This server calculates sequence conservation using the BLOSUM62 matrix with a similarity threshold of 0.7 for identical residues and 0.5 for similar residues. Conserved residues appear with a red background, whereas variable (mutated) residues appear with a white background.

The three-dimensional structure of the E2 protein of the YC-2025-Gansu2 strain was predicted using AlphaFold2 [30]. The amino acid sequence of the E2 protein was submitted to AlphaFold2, which performed multiple sequence alignment and structure prediction. Multiple predicted models were generated, and the confidence of each residue was assessed by the pLDDT (predicted local distance difference test) score. The model with the highest overall pLDDT score was selected as the final structure.

The three-dimensional structure of the E2 protein was visualized using PyMOL (The PyMOL Molecular Graphics System, version 2.5, Schrödinger, LLC (New York, NY, USA); available at: https://pymol.org, accessed on 3 April 2026) [31]. To map sequence conservation, the ESPript-generated PDB file containing conservation scores in the B-factor column was loaded into PyMOL. The protein surface was colored using the spectrum command based on B‑factor values, with red and orange highlighting the amino acid mutation sites.

#### 2.7.3. Glycosylation Site Analysis

Potential N-linked glycosylation sites were predicted using NetNGlyc 1.0 (https://services.healthtech.dtu.dk/service.php?NetNGlyc-1.0, accessed on 22 April 2026). Following the server’s default parameters, sites with a threshold score > 0.5 were considered potential N-glycosylation sites.

## 3. Results

### 3.1. Prevalence of BVDV in Gansu Province

**Figure 1:**
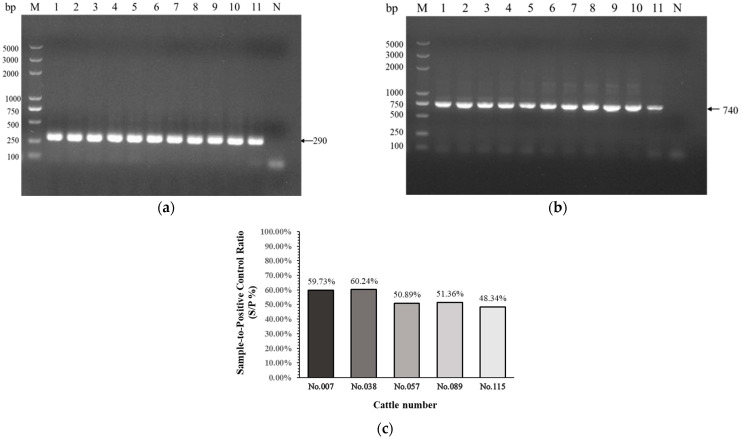
(**a**) Partial 5′UTR RT-PCR results; (**b**) Partial N^pro^ RT-PCR results; (**c**) Identification of BVDV Antigen in Bovine Positive Serum Samples. M: Trans2K Plus DNA Marker; N: Negative control; 1–11 Partial positive sample.

A total of 149 samples tested positive for BVDV by RT-PCR targeting the 5′UTR, producing the expected 290 bp band (Figure 1a). Sequencing confirmed all positive samples as BVDV. For a subset of positive samples, amplification of the N^pro^ gene yielded the expected 740 bp band (Figure 1b). Antigen testing showed that 5 of 128 serum samples were positive for BVDV antigen (Figure 1c). 

The overall prevalence in Gansu Province was 19.89% (95% CI: 17.07–22.71%), with notable regional variations. Jinchang City had the highest positivity rate at 33.33% (95% CI: 15.17–51.49%), followed by Wuwei City at 28.29% (95% CI: 22.83–33.75%), Gannan Prefecture at 27.94% (95% CI: 17.85–38.03%), Zhangye City at 21.43% (95% CI: 6.77–36.09%), and Jiuquan City at 20.69% (95% CI: 6.91–34.47%). Within Wuwei City, Tianzhu Tibetan Autonomous County had a positivity rate of 27.88%, accounting for 88.4% of the city’s samples and 89.0% of its positive cases. Other prefectures and cities, including Baiyin (16.67%, 95% CI: 5.83–27.51%), Pingliang (15.38%, 95% CI: 5.61–25.15%), Linxia (15.00%, 95% CI: 4.20–25.80%), Qingyang (13.33%, 95% CI: 3.45–23.21%), Lanzhou (13.33%, 95% CI: 2.15–24.51%), and Dingxi (11.54%, 95% CI: 4.73–18.35%), exhibited rates ranging from 11.54% to 16.67%. No BVDV-positive samples were detected in Jiayuguan, Tianshui, or Longnan (Table 2). Positive farms were mainly concentrated in the central region and the eastern part of the Hexi Corridor, indicating geographic clustering.

### 3.2. Subgenotype Distribution of BVDV in Gansu Province

**Figure 2:**
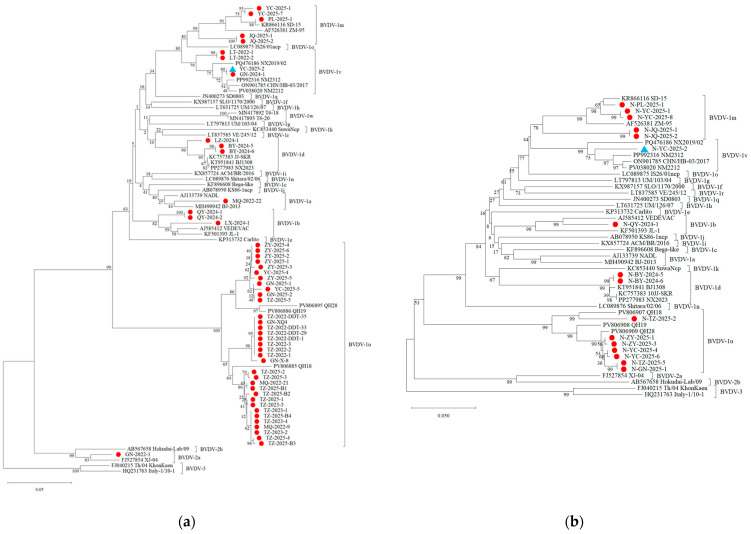
(**a**) Phylogenetic analysis based on 5′UTR; (**b**) Phylogenetic analysis based on N^pro^. ● and ▲ represent sequences from a subset of positive samples and isolated strains, respectively, based on 5′UTR in (**a**) and based on N^pro^ in (**b**).

A phylogenetic tree was constructed based on the 5′UTR sequences (Figure 2a). Among the 149 positive samples, 147 were classified as BVDV-1 and the remaining two as BVDV-2. Seven subtypes were identified, including BVDV-1a (n = 2), BVDV-1b (n = 12), BVDV-1d (n = 10), BVDV-1m (n = 15), BVDV-1v (n = 20), BVDV-1u (n = 88), and BVDV-2a (n = 2). BVDV-1u accounted for the highest proportion at 59.06% (95% CI: 51.14–66.98%), followed by BVDV-1v at 13.42%, BVDV-1m at 10.07%, BVDV-1b at 8.05%, and BVDV-1d at 6.72%. BVDV-1u was the predominant subtype circulating in Gansu Province. In Jinchang and Gannan, more than three subtypes co-circulated, indicating greater subtype diversity than in other areas (Table 3).

To validate the genotyping results, the N^pro^ gene was amplified from 59 samples, yielding 16 sequences. Subtype assignments from the N^pro^ sequences (Figure 2b) were consistent with those from the 5′UTR analysis (Figure 2a).

### 3.3. Characterization of the Isolated BVDV Strain

**Figure 3:**
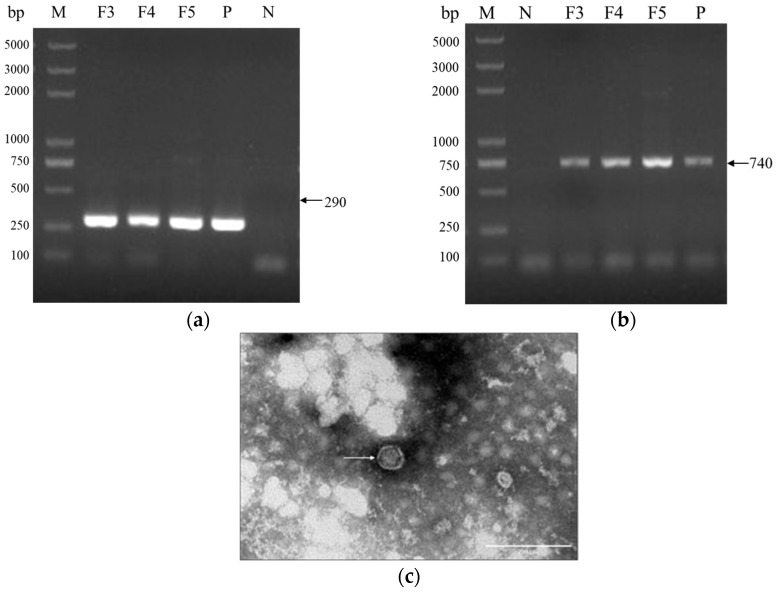
(**a**) 5′UTR RT-PCR identification results of BVDV; (**b**) N^pro^ RT-PCR identification results of BVDV; (**c**) Morphology of BVDV (30.0K×); M: Trans2K Plus DNA Marker; N: Negative control; P: Positive control; F3–F5: Samples of the third to fifth isolated strain.

**Figure 4:**
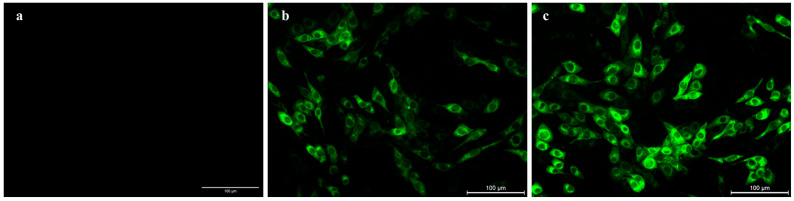
The isolated strain by IFA (400×) (**a**) MDBK cells as negative control; (**b**) MDBK cells infected with isolated strain; (**c**) Positive control.

The five antigen-positive serum samples were individually inoculated into MDBK cells, each followed by five consecutive blind passages. No cytopathic effects were observed during the five passages, suggesting that the isolated strain belongs to the non-cytopathic biotype. Viral cultures from passages 3, 4, and 5 all tested positive for BVDV, as confirmed by RT-PCR (Figure 3a,b). Electron microscopy of the fifth-passage virus culture revealed spherical viral particles approximately 40–60 nm in diameter with typical enveloped morphology (Figure 3c). Immunofluorescence staining of the fifth-passage virus culture revealed specific green fluorescence (Figure 4).

### 3.4. Complete Genome Analysis of the Isolated BVDV-1v Strain

The complete genome sequence of the isolated strain, designated YC-2025-Gansu2 and assigned GenBank accession number PV945812.1, was obtained by high-throughput sequencing. The total genome length was 12,129 bp. The open reading frame of this strain was 11,703 nt, encoding 3900 amino acids. The 5′UTR and 3′UTR were 287 nt and 140 nt in length, respectively, consistent with the typical genomic architecture of BVDV.

**Figure 5:**
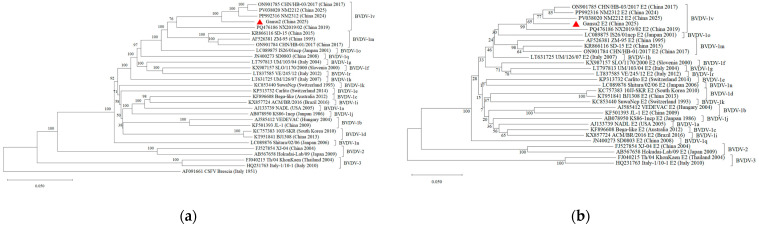
(**a**) Phylogenetic analysis based on BVDV whole genome sequences; (**b**) Phylogenetic tree constructed based on the amino acid sequence of E2 protein. ▲ represents sequences of isolated strains: whole-genome sequences in (**a**) and E2 amino acid sequences in (**b**).

The phylogenetic tree (Figure 5a) showed that the isolate clustered closely with BVDV-1v reference strains, including Inner Mongolia isolates MN2212 and MN2312 and Hubei isolate HB-03/2017. The nucleotide identity at the whole-genome level ranged from 94.71% to 94.96%, confirming its identification as a BVDV-1v subtype. In contrast, the isolate shared markedly lower nucleotide identity with other BVDV-1 subtypes (below 87%) and even lower identity with BVDV-2 and BVDV-3.

Amino acid sequence comparison (Figure 5b) showed that the YC-2025-Gansu2 isolate shared 96.3–96.5% amino acid identity with the BVDV-1v reference strains. In contrast, it shared less than 92% identity with other BVDV-1 reference strains.

Recombination analysis was conducted using RDP4 with eight detection methods: RDP, GENECONV, BootScan, MaxChi, Chimaera, SiScan, 3Seq, and Phylpro. Across the sequence dataset, 13 recombination events were identified. In most events, PQ476186 and PV038020 were detected as recombinant sequences, while AF526381, ON901785, and LC089875 served as major or minor parents. The YC-2025-Gansu2 strain was not identified as a recombinant or parental sequence in any of these 13 events, a finding consistently supported by all eight detection methods.

### 3.5. Molecular Characterization of the E2 Protein of the YC-2025-Gansu2 Strain

**Figure 6:**
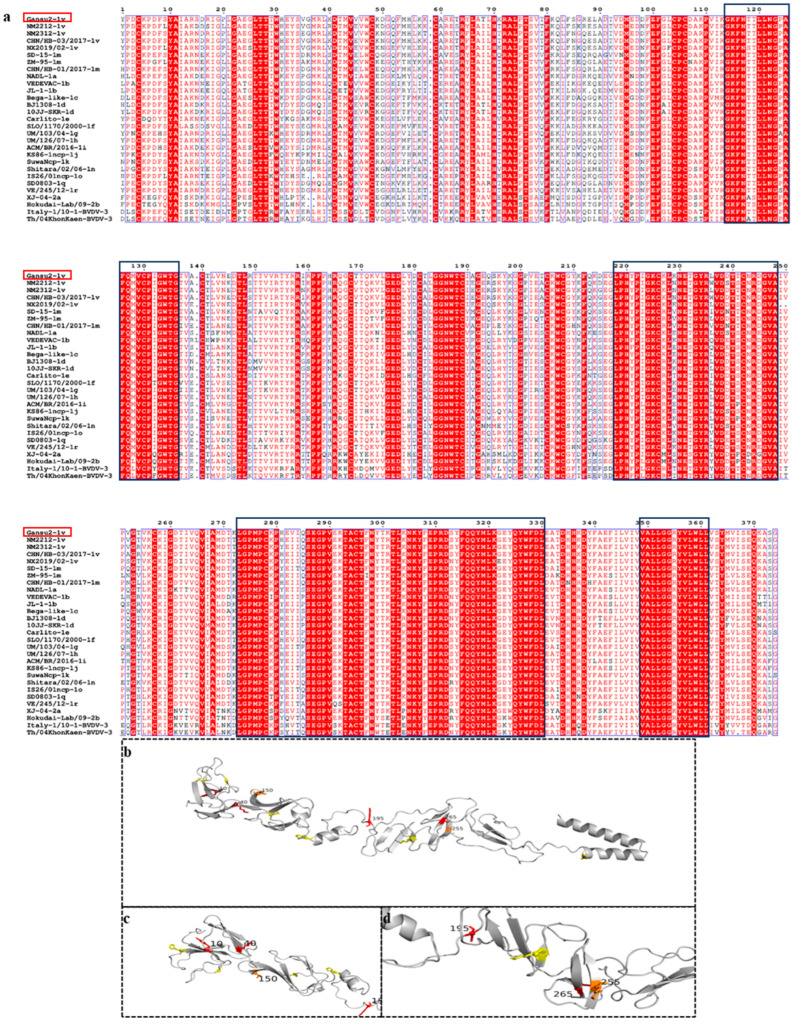
(**a**) Sequence alignment of BVDV E2 sequences; (**b**) Overall structure of the E2 protein of the YC-2025-Gansu2 strain; (**c**,**d**) Detailed view of the E2 protein structure. Amino acids are shown using single-letter codes. In panel (**a**): red boxes indicate the isolated target strain (YC-2025-Gansu2); black boxes indicate highly conserved regions after sequence alignment. Red letters highlight key residues; white letters on red background indicate functionally or structurally important positions; black letters on white background indicate non-conserved or less conserved positions.

To characterize E2 protein variation in the isolated strain, the E2 amino acid sequence of YC-2025-Gansu2 was aligned with 28 reference BVDV strains using ClustalW. ESPript-based visualization identified 12 distinct amino acid substitutions, all located within the hypervariable region, each altering either charge or polarity (Table 4). Four of these mutations at positions 10, 40, 195, and 265 fell within known neutralizing antibody-binding epitopes. In contrast, the four core conserved domains, spanning residues 114 to 136, 218 to 248, 273 to 330, and 349 to 361, remained highly preserved across all reference strains, with no insertions or deletions observed (Figure 6a). Compared with four BVDV-1v reference strains, Gansu2 had three distinct amino acid substitutions at positions 25, 40, and 265, of which site 265 lay within a neutralizing antibody-binding epitope. The remaining nine mutations represented cross-subtype variations. These findings suggest that Gansu2 is highly conserved within the 1v subtype while exhibiting considerable genetic distinctiveness in the broader BVDV sequence population.

PyMOL visualization of the E2 protein structure showed that the 12 mutation sites are predominantly located on the protein surface and at the interfaces of functional domains (Figure 6b). Sites 10, 40, 195, and 265, which lie within neutralizing antibody epitopes, are surface-exposed and capable of directly interacting with antibodies. Importantly, none of the mutations identified in this study compromise the core conserved domains of the E2 protein, thereby preserving its structural integrity and biological functionality.

To assess whether the amino acid substitutions affect N-glycosylation, N-glycosylation sites of the Gansu2 strain were predicted using NetNGlyc-1.0 with a threshold of 0.5. The Gansu2 strain E2 protein contains four putative N-glycosylation sites at positions 117 (NTTL, 0.6258), 186 (NWTC, 0.7167), 230 (NETG, 0.5732), and 298 (NYTK, 0.6221) (Table 5). These four sites were completely conserved in all BVDV-1v reference strains examined. Among the 28 reference strains, eight (KR866116, ON901784, KC757383, KT951841, LT631725, LT837585, FJ527854, and AB567658) had an additional N-glycosylation site, whereas the Gansu2 strain did not. These results indicate that the Gansu2 strain retains the conserved N-glycosylation pattern of BVDV-1 isolates, with no gain or loss of glycosylation sites.

## 4. Discussion

BVDV was first identified in North America in 1946 [32,33]. BVDV has a broad host range, infecting more than 40 animal species, including sheep, cattle, swine, and camels [34,35]. It was frequently co-infected with other pathogens, such as bovine coronavirus (BCoV), infectious bovine rhinotracheitis virus (IBRV), and *Pasteurella* spp., causing high fever, diarrhea, mucosal lesions, immunosuppression, and reproductive disorders [36,37,38]. Globally, BVDV-1 is the most prevalent genotype, with subtypes 1a, 1b, 1c, and 1m most frequently reported, particularly in Asia [11]. BVDV-2 has a worldwide distribution, with subtypes 2a and 2b identified in Shandong and Xinjiang, China [39,40]. In contrast, BVDV-3 is mainly detected in countries such as Brazil and Thailand [8,10,11], and the first documented occurrence in China was reported in 1980 [37]. In recent years, the highest prevalence rates in China have been observed in Fujian (90%), followed by Shaanxi (88.9%) and Shandong (83.3%) [12]. In this study, we analyzed 749 samples from 62 cattle farms across Gansu Province from 2021 to 2025, and the overall BVDV prevalence was 19.89%; this result is consistent with previously reported rates in northwestern China, ranging from 15% to 25% [12,15]. Marked regional variation was also observed, and the highest positive rate (33.33%) was found in Jinchang City, followed by Wuwei City (28.29%) and Gannan Prefecture (27.94%). No positive cases were detected in Jiayuguan, Tianshui, or Longnan. This geographical variation may be attributed to factors such as breeding density, livestock introduction frequency, biosecurity measures, and sampling distribution. Notably, positive cases in Tianzhu County, Wuwei City, were predominantly from white yak herds, suggesting sustained BVDV transmission within the yak population in this area.

Previous studies in northwest China have reported subtypes 1a, 1b, 1c, 1m, 1o, 1p, and 1q [15,41,42], some of which were also detected in our research. The most prevalent subtype in this study was BVDV-1u, accounting for 59.06% of cases, followed by BVDV-1v at 13.42%. Notably, the identification of novel subtypes 1v and 1u suggests cross-regional transmission and evolutionary dynamics of BVDV. Studies have shown that the nucleotide mutation rate in the coding region is higher during persistent infection than during acute infection [43,44]. Recently, the detection of novel subtypes such as BVDV-1v, 1u, and 1w in China indicates a capacity for rapid mutation and evolutionary diversification within BVDV subtypes. BVDV-1u, as the dominant subtype, is widely distributed across Gansu Province, which may be associated with regional cattle breeding practices, immunological selective pressure, and viral adaptive evolution.

Tianzhu County in Wuwei City, the main breeding area of white yaks, had a BVDV positivity rate of 28.29%, with the BVDV-1u subtype accounting for 97.26% of detected cases. Similarly, Gannan Prefecture, a yak breeding area on the eastern edge of the Qinghai–Tibet Plateau, had a positivity rate of 27.94%, with BVDV-1u accounting for 73.68% of infections. The consistent predominance of BVDV-1u in both yak-populated areas, despite their different geographical locations, suggests possible host specificity or a transmission advantage of this subtype in yaks. Several factors may contribute to the elevated BVDV prevalence in yaks, including frequent herd mixing due to traditional grazing practices, difficulties in eradicating persistently infected animals, and the absence of quarantine measures for newly introduced breeds. Notably, the positivity rate in Gannan Prefecture exceeded the provincial average, indicating a relatively severe BVDV epidemic in yak breeding areas.

In this study, the BVDV-1v strain YC-2025-Gansu2 was successfully isolated. Whole-genome and amino acid analyses confirmed its classification as BVDV-1v. The strain showed high genetic identity (94.71–94.96%) with isolates from Inner Mongolia and Hubei, but lower identity (88.22%) with isolates from Ningxia, suggesting regional genetic variation within the BVDV-1v subtype in China. The Gansu2 isolate exhibited nucleotide identities of 85.56–86.10% with 1m strains and 84.77–85.34% with 1o strains, higher than those with other BVDV-1 subtypes (79.58–82.74%), as well as with BVDV-2 and BVDV-3. These findings indicate that BVDV-1v shares a closer evolutionary relationship with subtypes 1m and 1o, consistent with Zhu et al. [7], who proposed that BVDV-1v may have originated from recombination between BVDV-1m and BVDV-1o. Phylogenetic analysis based on whole-genome and E2 amino acid sequences confirmed that Gansu2 clusters with BVDV-1v reference strains, adjacent to branches containing 1m and 1o subtypes, collectively forming a larger evolutionary clade. This topology aligns with Zhu et al. [7], suggesting a shared ancestral origin for these three subtypes. Notably, Gansu2 showed higher identity with 1m than with 1o, indicating a closer evolutionary relationship between 1m and 1v. Geographically, BVDV-1v, 1m, and 1o subtypes have been identified across multiple Chinese provinces. The 1m and 1o subtypes are widely prevalent in northwest China [42], while the 1v subtype has been consecutively detected in Inner Mongolia, Hubei, and Ningxia [7,45]. Their considerable genetic similarity and partially overlapping distributions suggest that BVDV-1v may have gradually evolved from 1m or 1o subtypes under selective pressures, or that recombination events may have occurred among them.

Both the 5′UTR and N^pro^ regions lie within conserved parts of the BVDV genome, but their amplification performance differs mainly because of amplicon length. As reported by Mahony et al. [46], the 5′UTR is surrounded by highly conserved motifs that allow easy amplification from various field samples, and its short length enables rapid and accurate sequence data acquisition. These features make the 5′UTR a good choice for initial phylogenetic analysis and for rapid genotyping of large numbers of viral samples. In contrast, the longer N^pro^ amplicon needs a more intact RNA template, so it is more likely to fail in clinical samples where viral RNA is partly degraded or viral loads are low. The reason is that shorter amplicons tolerate template fragmentation better. Even if RNA is randomly broken, only the primer binding sites need to remain for successful amplification. Thus, short amplicons have a higher chance of being amplified from a pool of degraded RNA fragments. In this study, the number of N^pro^ sequences obtained was 16 out of 59 samples, or 27.1 %, which was indeed lower than that of the 5′UTR (149 out of 749 samples, 19.89 %). Even so, the genotypes determined from the N^pro^ sequences matched those from the 5′UTR completely. This is consistent with Mahony et al. [46], who found that phylogenetic results from the 5′UTR and coding regions are similar. Therefore, the 5′UTR short amplicon is well suited for detecting low-level BVDV infections and for large-scale rapid diagnostic use.

At the amino acid level, Gansu2 showed 87.2–88.5% identity with the E2 protein of 1m subtypes and 86.5–87.3% identity with 1o subtypes. Although lower than its identity with the 1v subtype (96.3–96.5%), this level remains substantially higher than that observed with other subtypes. The relatively high homology between Gansu2 and the E2 proteins of 1m and 1o subtypes suggests that these three subtypes may share comparable antigenic properties, providing an important reference for vaccine strain selection and cross-protection assessment.

Recombination is an important evolutionary mechanism for RNA viruses, potentially leading to changes in antigenicity, pathogenicity, and host tropism [47]. In this study, RDP4 analysis of the BVDV complete genome detected 13 recombination events across the sequence dataset. PQ476186 and PV038020 were identified as recombinant sequences, while AF526381, ON901785, and LC089875 served as major or minor parents. The YC-2025-Gansu2 strain was not involved in any recombination event, supporting its use as a non-recombinant reference sequence for phylogenetic analyses. Notably, BVDV-1v is an emerging genotype whose origin may involve inter-genotype recombination [7]; however, the absence of recombination signals in Gansu2 suggests that even within the same subtype, different strains can have distinct recombination histories. Thus, Gansu2 represents an evolutionary lineage within BVDV-1v that has not undergone significant recombination, making it a valuable non-recombinant reference for comparative evolutionary analyses with other recombinant strains of this subtype. Our findings further confirm the overall conservation of the BVDV genome in evolution.

The E2 protein is the primary target for neutralizing antibodies induced by BVDV. Amino acid variations in this protein are closely associated with immune evasion and geographic adaptation [48]. During persistent infection and vertical transmission, mutations tend to accumulate preferentially within the E2 coding region [43,44]. In this study, the Gansu2 isolate carried twelve distinct amino acid substitutions in the E2 protein, all located within the hypervariable region. Each mutation involved changes in either charge or polarity. Four of these mutations at positions 10, 40, 195, and 265 fell within established neutralizing antibody-binding epitopes. The predicted three-dimensional structure showed that the twelve mutation sites are predominantly located on the protein surface and at the interfaces of functional domains. The four epitope sites are surface-exposed and capable of directly interacting with antibodies.

N-glycosylation analysis revealed that the Gansu2 strain contains four conserved N-glycosylation sites at positions 117, 186, 230, and 298, which were completely conserved in all BVDV-1v reference strains. Notably, eight of the 28 reference strains possessed an additional fifth N-glycosylation site, belonging to diverse genetic backgrounds including BVDV-1m, BVDV-1d, BVDV-1i, BVDV-1q, BVDV-1n, and BVDV-2a. This observation is consistent with a previous report on the SD-15 strain (BVDV-1m), which also harbors an extra N-glycosylation site at position 240 [49]. The presence of an additional glycosylation site across multiple subtypes suggests that this feature is widely distributed among different BVDV lineages. In contrast, the Gansu2 strain did not acquire this extra site, indicating that its antigenic variation is primarily attributable to direct amino acid substitutions within epitope regions rather than alterations in glycosylation patterns.

## 5. Conclusions

This study revealed the epidemiological status of BVDV in Gansu Province. Seven subtypes were identified, with BVDV-1u being predominant. A BVDV-1v strain, YC-2025-Gansu2, was isolated, and its complete genome was sequenced. Phylogenetic analysis indicated that this strain occupies a relatively distinct evolutionary position within the 1v subtype. These findings enrich the genetic subtype database of BVDV in this region and provide a scientific basis for its prevention, control, and evolutionary research.

## Figures and Tables

**Table 1:** BVDV 5′UTR and N^pro^ Amplified Primer Sequences.

Primer Names	Primer Sequences(5′-3′)	Fragment Length (bp)
BVDV 5′UTR-F	CTAGCCATGCCCTTAGTAGGACTA	290
BVDV 5′UTR-R	CAACTCCATGTGCCATGTACAGCA	
BVDV N^pro^-F	TCTCTGCTGTACATGGCACATG	740
BVDV N^pro^-R	TTGTTRTGGTACARRCCGTC	

**Table 2:** Detection results in prefecture-level cities across Gansu Province.

Administrative Regions	Sample Type	Samples	BVDV Positive Samples	BVDV Positive Rate	Total Samples	Total Positive Samples	Total Positive Rate
Wuwei	serum	256	71	27.73%	258	73	28.29% (95% CI: 22.83–33.75%)
	spleen	2	2	100%			
Jiuquan	serum	25	2	8%	29	6	20.69% (95% CI: 6.91–34.47%)
	nasal swab	2	2	100%			
	small intestine	2	2	100%			
Zhangye	serum	20	0	0%	28	6	21.43% (95% CI: 6.77–36.09%)
	feces	8	6	75%			
Jiayuguan	serum	12	0	0%	12	0	0%
Jinchang	serum	24	8	33.33%	24	8	33.33% (95% CI: 15.17–51.49%)
Baiyin	serum	36	6	16.67%	36	6	16.67% (95% CI: 5.83–27.51%)
Linxia	serum	40	6	15%	40	6	15% (95% CI: 4.20–25.80%)
Dingxi	serum	78	9	11.54%	78	9	11.54% (95% CI: 4.73–18.35%)
Qingyang	serum	45	6	13.33%	45	6	13.33% (95% CI: 3.45–23.21%)
Tianshui	serum	42	0	0%	50	0	0%
	nasal swab	8	0	0%			
Longnan	serum	12	0	0%	12	0	0%
Pingliang	serum	35	4	11.43%	39	6	15.38% (95% CI: 5.61–25.15%)
	nasal swab	4	2	50%			
Gannan	serum	58	17	29.31%	68	19	27.94% (95% CI: 17.85–38.03%)
	feces	10	2	20%			
Lanzhou	serum	30	4	13.33%	30	4	13.33% (95% CI: 2.15–24.51%)
total	serum	713	133	18.65%	749	149	19.89% (95% CI: 17.07–22.71%)
	feces	18	8	44.44%			
	spleen	2	2	100%			
	nasal swab	14	4	28.57%			
	small intestine	2	2	100%			

**Table 3:** Prevalence of BVDV subtypes in cattle farms in Gansu Province from 2021 to 2025.

Subgenotype (n)	City/Prefecture	Representative Strain	Number of Sequences (n)	Proportion (%)
BVDV-1u (n = 88)	Wuwei	MQ-2022-9	13	59.06% (95% CI: 51.14–66.98%)
	Wuwei	TZ-2022-1	6	
	Wuwei	TZ-2022-DDT-29	30	
	Wuwei	TZ-2023-1	6	
	Wuwei	TZ-2025-1	4	
	Wuwei	TZ-2025-B1	12	
	Jinchang	YC-2025-4	2	
	Zhangye	ZY-2025-1	6	
	Gannan	GN-XQ4	4	
	Gannan	GN-X-8	8	
	Gannan	GN-2025-1	2	
BVDV-1v (n = 20)	Jinchang	YC-2025-2	3	13.42% (95% CI: 7.87–18.97%)
	Dingxi	LT-2022-1	9	
	Gannan	GN-2024-1	3	
BVDV-1m (n = 15)	Jiuquan	JQ-2025-1	6	10.07% (95% CI: 5.19–14.95%)
	Jinchang	YC-2025-1	3	
	Pingliang	PL-2025-1	6	
BVDV-1b (n = 12)	Linxia	LX-2024-1	6	8.05% (95% CI: 3.64–12.46%)
	Qingyang	QY-2024-1	6	
BVDV-1d (n = 10)	Baiyin	BY-2024-5	6	6.72% (95% CI: 2.66–10.78%)
	Lanzhou	LZ-2024-1	4	
BVDV-1a (n = 2)	Wuwei	MQ-2022-22	2	1.34% (95% CI: 0–3.19%)
BVDV-2a (n = 2)	Gannan	GN-2022-1	2	1.34% (95% CI: 0–3.19%)
Total	/	/	149	100%

**Table 4:** Analysis of amino acid mutation sites in E2 protein of YC-2025-Gansu2 strain.

Mutation Site	Gansu2 Strain Amino Acid	Amino Acid in Other Strains	Substitution Type	Functional Region Annotation
10	K(Lys)	R, P, Q	Polar → Polar (charge change)	N-terminal surface-exposed loop located within an antigenic epitope region
25	A(Ala)	V, T, S	Nonpolar → Polar/Nonpolar	N-terminal flexible loop involved in antigenic variation
40	D(Asp)	E, G, N	Polar → Polar (charge change)	Antibody-binding and immune recognition region
70	S(Ser)	P, A, T	Polar → Nonpolar/Polar	Surface-exposed loop within a B-cell antigenic epitope region
95	F(Phe)	L, V, I	Nonpolar → Nonpolar	N-terminal edge within an antigenic variation hotspot
150	A(Ala)	V, Q, T	Nonpolar → Polar/Nonpolar	DB-DC domain connecting loop
170	T(Thr)	S, A, N	Polar → Polar/Nonpolar	Central antigenic epitope-enriched region associated with immune response
195	D(Asp)	E, G, N	Polar → Polar (charge change)	Surface-exposed loop within the core region of a B-cell epitope
210	P(Pro)	S, A, T	Nonpolar → Polar/Nonpolar	Dominant antigenic epitope region serving as a host immune recognition site
255	S(Ser)	T, A, G	Polar → Polar/Nonpolar	DC-DD domain flexible loop involved in protein conformation regulation
265	N(Asn)	D, E, Q	Polar → Polar (charge change)	Surface antigenic loop serving as a key site for immune escape
340	A(Ala)	S, T, V	Nonpolar → Polar/Nonpolar	C-terminal near-membrane exposed loop, antigenicity-related region

**Table 5:** Predicted N-glycosylation sites in the E2 protein of the YC-2025-Gansu2 strain.

Position	Motif	Potential	Jury Agreement	Result
117	NTTL	0.6258	7/9	+
186	NWTC	0.7167	9/9	++
230	NETG	0.5732	6/9	+
298	NYTK	0.6221	7/9	+

+, potential N-glycosylation site; ++, highly potential N-glycosylation site.

## Data Availability

The dataset presented in this study is openly available in the GenBank database (https://www.ncbi.nlm.nih.gov/genbank/). The accession number is PV945812.1.
